# Cardol triene inhibits dengue infectivity by targeting kl loops and preventing envelope fusion

**DOI:** 10.1038/s41598-018-35035-w

**Published:** 2018-11-09

**Authors:** Parichat Kanyaboon, Thanaphon Saelee, Aphinya Suroengrit, Kowit Hengphasatporn, Thanyada Rungrotmongkol, Warinthorn Chavasiri, Siwaporn Boonyasuppayakorn

**Affiliations:** 1 Applied Medical Virology Research Unit, Department of Microbiology, Faculty of Medicine, Chulalongkorn University, Bangkok, 10330 Thailand; 2 Medical Microbiology, Interdisciplinary Program, Graduate School, Chulalongkorn University, Bangkok, 10330 Thailand; 3 Department of Biology, Faculty of Science, Chulalongkorn University, Bangkok, 10330 Thailand; 4 Bioinformatics and Computational Biology Program, Graduated School, Chulalongkorn University, Bangkok, 10330 Thailand; 5 Structural and Computational Biology Research Group, Department of Biochemistry, Faculty of Science, Chulalongkorn University, Bangkok, 10330 Thailand; 6 Center of Excellence in Natural Products Chemistry, Department of Chemistry, Faculty of Science, Chulalongkorn University, Bangkok, 10330 Thailand

## Abstract

Dengue virus causes a global burden that specific chemotherapy has not been established. A previous report suggested that anacardic acid inhibited hepatitis C virus infection. Here, we explored structure activity relationship of anacardic acid, cardanol, and cardol homologues with anti-DENV cellular infectivities. Cardol triene showed the highest therapeutic index at 29.07 with the CC_50_ and EC_50_ of 207.30 ± 5.24 and 7.13 ± 0.72 µM, respectively. Moreover, we observed that the more unsaturated the hydrocarbon tail, the higher the CC_50_s in all head groups. High CC_50_s were also found in HepG-2, THP-1, and HEK-293 cell lines where cardol triene CC_50_s were 140.27 ± 8.44, 129.77 ± 12.08, and 92.80 ± 3.93 µM, respectively. Cardol triene expressed pan-dengue inhibition with the EC_50_s of 5.35 to 8.89 µM and kl loops of dengue envelope proteins were major targets. The strong binding energy at T48, E49, A50, P53, K128, V130, L135, M196, L198, Q200, W206, L207, I270, and L277 prevented cellular pH-dependent fusion. Zika virus kl loops were aligned in the closed position preventing cardol triene to bind and inhibit fusion and infectivity. This study showed for the first time that cardol triene had a potential for further development as anti-dengue inhibitors.

## Introduction

Dengue infection causes a burden over worldwide tropicals with 390 million infections per year^1^. Of these statistics, Asia represented approximately 75% of the total reports^2^. Dengue virus belongs to the family *Flaviviridae*, genus *Flavivirus*, and consists of four serotype (DENV1-4). The infectious virion contains lipid envelope surrounding an icosahedral nucleocapsid. The nucleocapsid contains an 11 kilobases, single stranded, positive sense RNA. Envelope (E) protein is an immunogen and a major antigenic cross-reactive agent^3^. All serotypes of dengue viruses are transmitted by *Aedes* mosquitoes^2^. Severe clinical manifestations including plasma leakage, hemorrhage, and shock are caused by robust but incompetent immunological responses to secondary heterotypic infection^4,5^. Remarkably, the antibodies responding to those secondary heterotypic infection are unable to perform complete neutralization and the antibody-tagged viruses are opsonized by macrophage. The viral replication is enhanced inside the cell resulting in increasing the risk to severe dengue development^6^. Moreover, it was clearly showed that the correlation existed between high viral load, prolonged viremia and clinical severity^6–8^. Therefore, reducing the viral load should be a direct means to treat acute dengue infection in order to reduce the progression to severe clinical outcomes.

Antiviral drug discovery and development have been substantially advanced within a few decades. Five active leads have entered the clinical trials^9–13^ but none of them has been launched as a licensed drug yet. Natural products derived from medicinal plants are rich source of potential antiviral inhibitors^14^. Phenolic lipids are amphiphilic molecules containing phenolic rings and long aliphatic chains. Recently, accumulated evidences suggested that phenolic lipids showed a broad spectrum antimicrobials^15^, and anti-cancer^16^ activities by disturbing the phospholipid membrane^17^ and inducing apoptosis^16,18^. One of the major sources of phenolic lipids was cashew nut shell liquid (CNSL) existing as mixture of anacardic acid, cardanol, and cardol head groups and saturated (C15:0), monoene (C15:1), diene (C15:2), and triene (C15:3) tails. There is a previous study describing saturated anacardic acid (C15:0) as an inhibitor of hepatitis C virus by interfering with the viral entry, translation, replication, and release^19^. Dengue virus and hepatitis c virus are likewise members of the family *Flaviviridae*, therefore it is possible that phenolic lipids should also effectively inhibit dengue and Zika viruses. Since there is limited knowledge of phenolic lipids as antivirals, we aimed to characterize the efficacies of all homologues of CNSL-derived phenolic lipids for dengue virus inhibition. Moreover, the potential candidates would be studied for activities against dengue virus serotype 1–4 and Zika virus; as well as cytotoxicity to human-derived cell lines. The mechanism of drug action and molecular target would also be explored to bring insights into further lead optimization on potential targets.

## Results

### Preliminary results of phenolic lipids as DENV2 inhibitors

**Figure 1:**
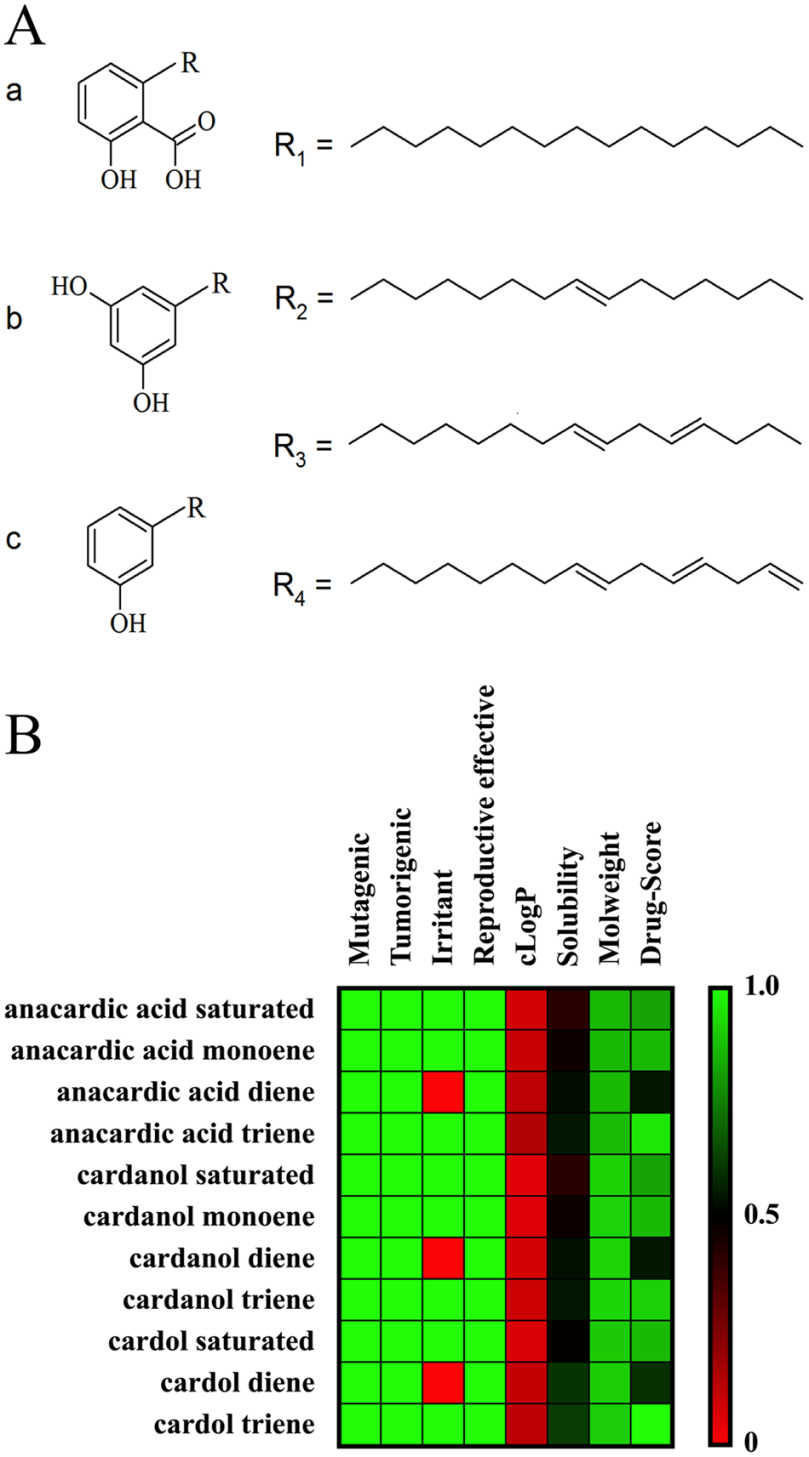
Chemical structure and computational screening. (**A**) Phenolic lipid structures consisting of three head groups a) anacardic acid, b) cardol, c) cardanol; and the C15 hydrocarbon tails R1) saturated, R2) monoene, R3) diene, and R4) triene. (**B**) Computational screening for drug likeness using Molecular Property Explorer and RTECS database.

**Figure 2:**
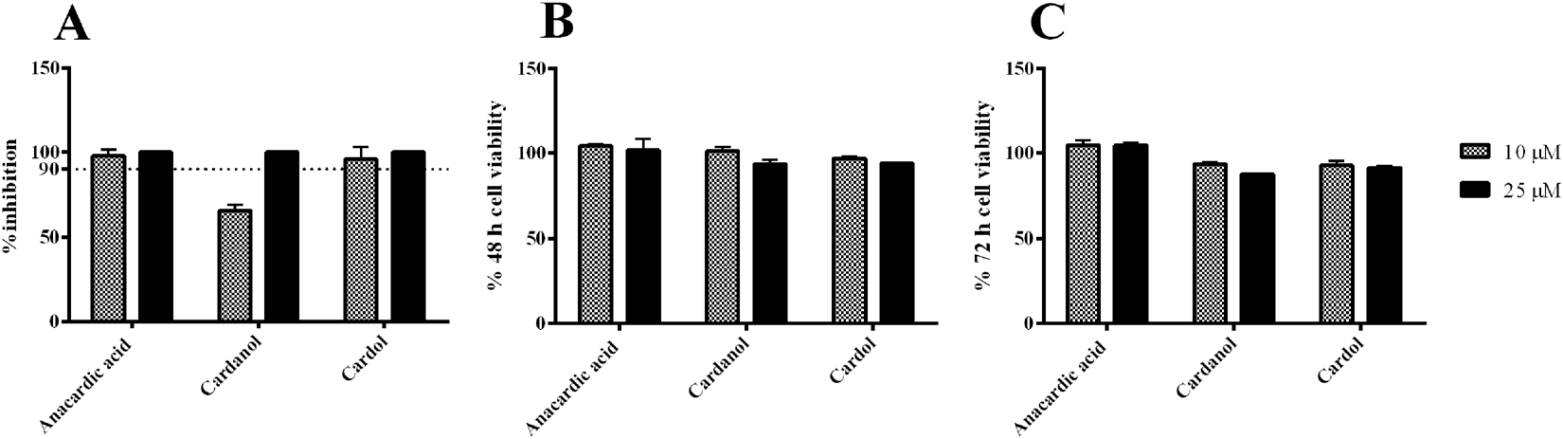
Preliminary cell-based screening. (**A**) DENV2 inhibition; Vero (5 × 10^4^ cells) were treated with DENV2 (M.O.I. of 0.1) and 10 and 25 µM anacardic acid, cardanol, and cardol mixtures for 72 h and supernatants were analyzed by plaque titration. DMSO was used as a mock treatment referring to 100% infection. (**B**,**C**) Cytotoxicity of Vero cells at (**B**) 48 h and (**C**) 72 h were performed by treatment of Vero (5 × 10^4^ cells) with 10 and 25 µM anacardic acid, cardanol, and cardol mixtures for 48 and 72 h. Cell viability was accessed using MTS reagents and DMSO-treated cells referred to 100% cell survival.

A previous report suggested that anacardic acid mixture extracted from leaves of *Gingko bilob*a inhibited several steps of hepatitis C virus replication^19^. Hepatitis C virus is one of the members of the family *Flaviviridae*, therefore, it is likely that anacardic acids, cardanol, and cardol should inhibit dengue virus replication. We extracted phenolic lipids from cashew nut shell liquid (CNSL) and primarily characterized by the three head groups into anacardic acid, cardanol, and cardol (Fig. 1A). Each compound was first screened for druglikeness using two pharmacological characters and four toxicity risks listed in materials and methods. Results showed that trienes from all phenolic heads scored the highest, whereas dienes scored the lowest regarding from the irritant factor (Fig. 1B). Moreover, anacardic acid, cardanol and cardol mixtures were co-incubated with DENV2 infected Vero cells (M.O.I. of 0.1) at 10 µM and 25 µM for 72 h and the viral inhibition was quantified by plaque titration assay. This preliminary data suggested that DENV2 virions were inhibited at 97.95, 65.30, and 95.91% when incubated with anacardic acid, cardanol and cardol at 10 µM, respectively (Fig. 2A); whereas the DENV2 virions were potently inhibited ( > 99%) by all compounds at 25 µM (Fig. 2A). Moreover, the viability of Vero cells were accessed in the presence of each compound at 10 and 25 µM for 48 and 72 h (Fig. 2B-C) and cytotoxicity results were less than 20 percent in all experimental conditions. Taken together, our preliminary data showed that anacardic acid, cardanol, and cardol inhibited DENV2 infectivity similar to that of HCV previously reported^19^.

### Cytotoxicity of phenolic lipid homologues

**Figure 3:**
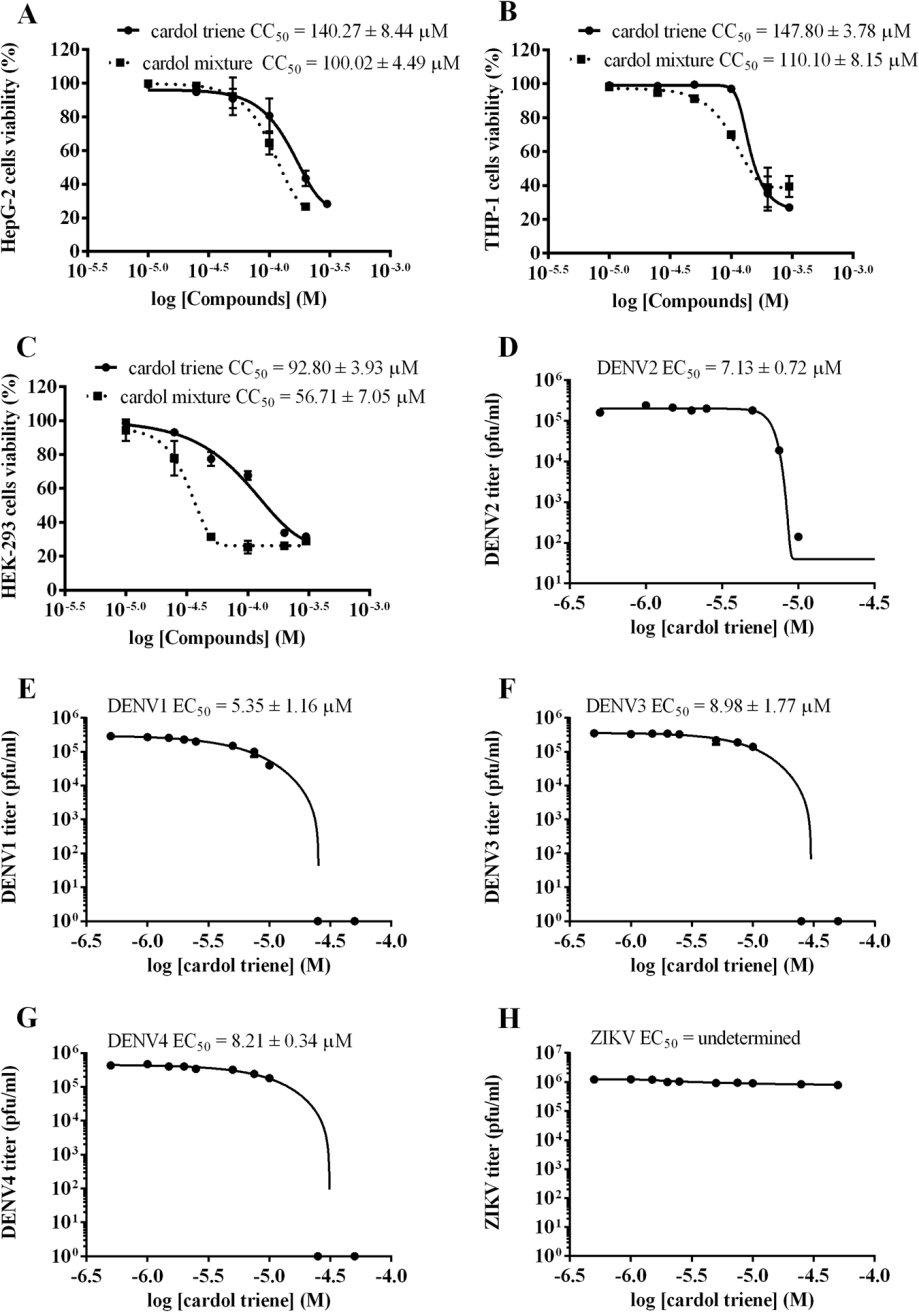
CC_50_s and EC_50_s of cardol triene. (**A**–**C**) Non-linear regression curves and calculated cytotoxicity concentrations (CC_50_s) of cardol triene (bold line) and cardol mixture (dash line) in (**A**) HepG2, (**B**) THP-1, (**C**) HEK-293 cell lines were shown. (**D**–**H**) Non-linear regression curves and calculated effective concentrations (EC_50_s) of cardol triene in Vero cells infected with (**D**) DENV2 (**E**) DENV1 (**F**) DENV3 (**G**) DENV4 and (**H**) ZIKV were shown. Graphs represented one of the independent experiments and values indicated means ± standard error of the means (SEM) from three independent experiments.

Next, the compound mixture were further purified and synthesized into saturated (C15:0), monoene (C15:1), diene (C15:2), and triene (C15:3) as described in materials and methods. Toxicities were analyzed and results were calculated to percent viability and CC_50_ were analyzed using non-linear regression curve (Table 1). Interestingly, anacardic and cardol trienes, had the highest CC_50_s at 115.13 ± 14.12, and 207.3 ± 5.24 µM, comparing with other homologues from the same head group. As the hydrocarbon tail became more saturated, the CC_50_ gradually decreased until reaching the lowest value at saturation (C15:0). Cardanol triene was insoluble in DMSO, therefore the compound was excluded from this study. It could be inferred that the polyunsaturated hydrocarbon may involve in stabilizing the cell membrane. Moreover, CC_50_s of the mixtures tend to follow those of the saturated homologues suggesting that the presence of saturated homologues, regardless of the proportion, could dominate the cell membrane disruption. Moreover, we further explored the CC_50_ of cardol triene in other cell lines to verify its protective effect. Three human-derived cell lines (THP-1, HEK-293, and HepG2) were used to co-incubate with cardol triene and cardol mixture (73% diene and 27% triene). Results showed that CC_50_s of cardol triene were about 30–50 µM higher than cardol mixture in all cell lines (Fig. 3A–C). In conclusion, cardol triene showed a distinctive CC_50_ value comparing with other homologues towards four distinctive mammalian cell lines.

**Table 1:** EC_50_^1^, CC_50_^2^, and TI^3^ of CNSL-derived phenolic lipids.

Compounds	EC_50_ (µM)**	CC_50_ (μM)**	TI
**Anacardic acid mixture**	**4.82 ± 1.71**	**58.80 ± 2.91**	**12.19**
Anacardic acid saturated	4.31 ± 1.17	66.33 ± 1.58	15.39
Anacardic acid monoene	12.59 ± 0.84	74.00 ± 0.85	5.88
Anacardic acid diene	Not inhibited	112.17 ± 8.57	—
Anacardic acid triene	7.48 ± 2.14	115.13 ± 14.12	15.38
**Cardanol mixture**	**11.06 ± 0.40**	**46.86 ± 2.94**	**4.23**
Cardanol saturated	5.89 ± 2.83	43.51 ± 1.10	7.39
Cardanol monoene	7.65 ± 2.58	98.70 ± 3.16	12.90
Cardanol diene	Not inhibited	159.40 ± 7.41	—
Cardanol triene*	—	—	—
**Cardol mixture**	**3.24 ± 0.51**	**60.51 ± 4.94**	**18.67**
Cardol saturated	12.72 ± 0.67	58.75 ± 0.43°	4.62
Cardol diene	11.90 ± 0.82	71.66 ± 5.27°	6.02
Cardol triene	7.13 ± 0.72	207.30 ± 5.24	29.07

^1^Effective concentration of indicated phenolic lipid to DENV2 NGC (M.O.I. of 0.1) infected Vero cells.

^2^Cytotoxic concentration of indicated phenolic lipid to Vero cells.

^3^Therapeutic index (TI, CC_50_/EC_50_).

*Insoluble.

**Data represented means ± standard error of the means (SEM) from three independent experiments, unless otherwise indicated.

°Data represented means ± standard error of the means (SEM) from two independent experiments due to limited compound availability.

### Efficacies of phenolic lipid homologues

Next, we studied the efficacies (EC_50_) of the phenolic lipid homologues against DENV2 infectivity in Vero cells. Briefly, compounds at 10 different concentrations were added to DENV2-infected Vero cells (M.O.I. of 0.1) during and post infection and the viral inhibition was determined by plaque titration. Efficacies of anacardic acids varied among homologues so that the structure-activity relationship was inconclusive. The most efficient anacardic acid was the saturated homologue and its result was similar to that of the mixture although only 18% of saturated anacardic acid was found in the total mixture. It is possible that the viral inhibition and cellular response might be predominately influenced by saturated anacardic acid regardless of the percent constituent. Noted that anacardic acids were major components of CNSL extracts and all homologues were purified directly from CNSL itself. However, CC_50_s of anacardic acids were generally lower than those of cardols (Table 1), therefore they were not chosen for further characterization.

Naturally derived cardanol mixture consisted of monoene, diene, and triene hydrocarbon species. The saturated cardanol was later synthesized by hydrogenation. Overall, the efficacy of cardanols were less potent than those of anacardic acids and cardols (Table 1). One of the homologues, cardanol triene, was insoluble in DMSO as previously described, therefore it was excluded from the study. Efficacy of cardanol mixture fell between those of the monoene and diene, suggesting that both homologues contributed to the viral inhibition. Interestingly, hydrogenation of cardanol potentiated the efficacy (EC_50_) to 5.89 ± 2.83 µM. However, cardanols were the most cytotoxic of all head groups, thus resulted in the least TI values. The group were then excluded from further investigations.

Last, cardol mixture and homologues were analyzed for their efficacies using the DENV2 infected cells previously described. Cardol mixture was the most potent DENV2 inhibitor beyond any caldol homologue with the EC_50_ of 3.24 ± 0.51 µM, and therapeutic index (TI) of 18.63 (Table 1), suggesting a synergistic effect from cardol diene and triene residing in the mixture. Analysis of the homologue showed that increasing the tail unsaturation correlated with increasing the viral inhibition. Cardol triene was the most effective at EC_50_ of 7.13 ± 0.72 µM, and therapeutic index (TI) of 29.07 (Table 1, Fig. 3D). Considering the TI values, the triene homologues showed the broadest spectrum suggesting strong candidates for further investigation. Moreover, the cell morphology under the treatment of DENV2 (M.O.I. of 1 and 5) and cardols were observed at 2, 24, and 48 h after infection and no apparent cytotoxicity were observed in any condition (Supplementary 1). From these results, cardol triene was selected for subsequent studies to verify the drug mechanism.

We further explored whether cardol triene would exhibit broad spectrum inhibition against dengue virus serotype 1–4 and Zika virus. Efficacies against DENV1 (16007), DENV3 (16562), DENV4 (c0036), and ZIKV (sv0010/15) (Fig. 3E–H) suggested that cardol triene similarly inhibited all dengue viruses, but did not inhibit Zika virus. Since dengue and Zika viruses are closely related, their protein structures and their mechanisms of viral replication were highly similar. Therefore, the character of potential targets were supposed to be conserved exclusively within dengue virus, but not in Zika virus. Investigating for the discrepancies between mechanism of dengue and zika virus replication, as well as between the two viral proteins, could result in identification of the molecular targets.

### Screening for possible targets of viral inhibition

**Figure 4:**
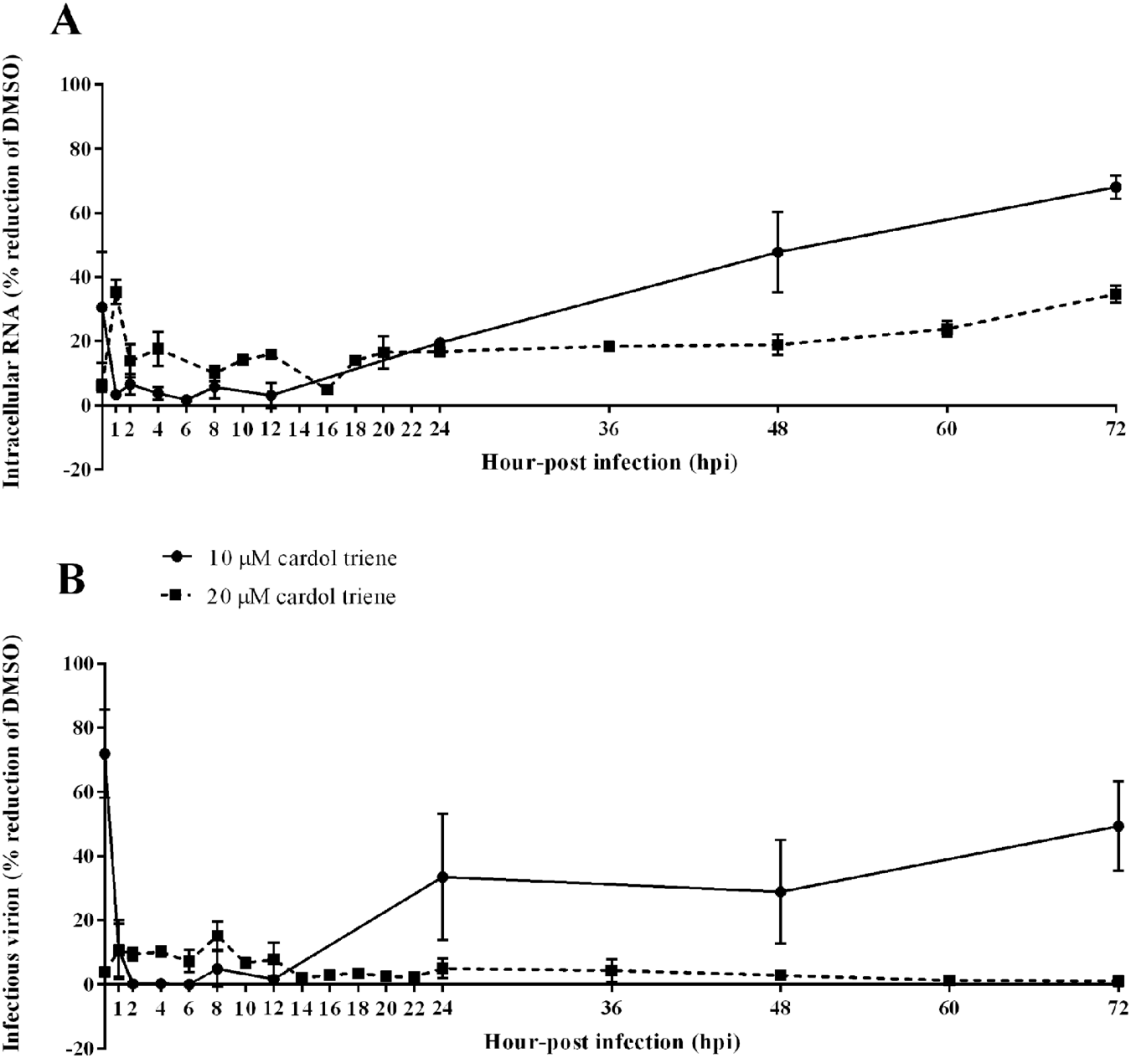
Time-of-drug-addition (TOA) study. DENV2 infected Vero cells were treated with 10 (bold line) and 20 (dash line) µM cardol triene at respective time-points after infection (hpi) and incubated for 72 h. Cell lysates and culture supernatants were collected for analysis by RT-qPCR and plaque titrations for (**A**) DENV2 intracellular RNA and (**B**) infectious virion, respectively.

Time of drug addition assay is a standard method to primarily identify the possible target of a newly characterized compound^20^. Based on the previous efficacy results, cardol triene was chosen as a representative of all CNSL-derived phenolic lipids. Cardol triene was prepared in DMSO and added to the final concentrations of 10 µM and 20 µM to DENV2 infected Vero cells (M.O.I. of 0.1). The compound was added to the cells at different time-points. DMSO alone was added to the infected cells as a mock treatment. After 72 h incubation, cell lysates and supernatants were collected for analysis of intracellular RNAs and infectious virions by RT-qPCR and plaque titration, respectively. Results showed that intracellular RNAs (Fig. 4A) and infectious virions (Fig. 4B) decreased immediately at 1 h and persisted until 48 h after infections suggesting that the compound globally inhibited the virus at early and late stages of the life cycle. Noted that the EC_50_ of cardol triene was 7.13 ± 0.72 µM; therefore, the viral replication should be suppressed beyond 50 percent by plaque titration under the treatment of cardol triene at both concentrations. From these results, we hypothesized that the phenolic compounds could bind to multiple targets at both early and late stages of the virus life cycle, similar to a previous report on HCV^19^.

### Cardol triene bound to kl loops and inhibited DENV fusion

**Figure 5:**
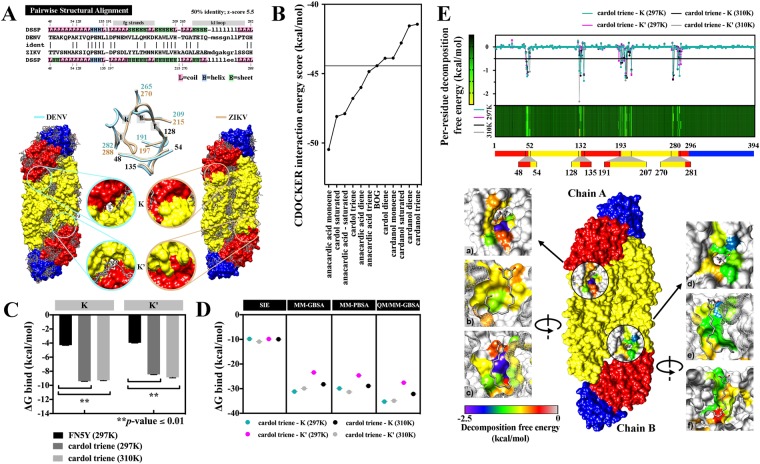
Molecular docking and MD simulation. (**A**) Structural comparison between DENV and ZIKV E proteins at kl loops. (**B**) Molecular docking of phenolic lipids and β-OG at kl hydrophobic pockets of DENV E dimer. (**C**–**E**) Structural dynamics of cardol triene to K and K’ sites of DENV E dimers. (**C**) Total binding free energy (ΔG bind) of cardol triene and K and K’ sites at 60–100 ns-trajectories at 297 and 310 Kelvin were compared with FN5Y at 297 K using SIE method. (**D**) Total binding free energy (ΔG bind) of cardol triene and K and K’ sites at 200–300 ns-trajectories at 297 and 310 Kelvin generated from SIE, MM-GBSA, MM-PBSA, and QM/MM-GBSA were compared. (**E**) Binding regions and residues were revealed as follows; 48–54 (T48, E49, A50, P53), 128–135 (K128, V130, L135), 191–207 (M196, L198, Q200, W206, L207), and 270–281 (I270, and L277).

The early life cycle of DENV mainly involved attachment, entry, and fusion. Due to the fact that cardol triene did not inhibit ZIKV, the close relatives of DENV; it is most likely that the drug targets were present only in DENV proteins and absent in those of ZIKV. Dengue and Zika envelope (E) proteins were first tested using blind docking technique. The cardol triene was preliminarily docked into DENV and ZIKV E dimers in order to detect the differences of ligand binding sites (Fig. 5A). Pairwise structural alignment at β-OG pocket revealed 50% identity between DENV and ZIKV with z-score of 5.5 (Fig. 5A). The β-OG pocket consists of four protein fragments including 48–54, 128–135, 191–209, and 265–282 in DENV, and 48–54, 128–135, 197–215 and 270–288 in ZIKV. The major difference was indicated to open and closed positioning of the kl loop, 265–282 in DENV and 270–288 in ZIKV. Also, blind docking results suggested that cardol triene was able to bind exclusively into dengue β-OG pocket (Fig. 5A). Molecular docking by CDOCKER was also used to analyze other phenolic lipids interacting at the kl loop pockets of DENV E dimer (Fig. 5B). The interaction energy scored between −41.44 to −50.47 kcal/mol and a β-OG crystal ligand was listed at 44.43 kcal/mol.

MD simulation was setup using the initial cardol triene/DENV E complex structure adopted from the best orientation and interaction energy score calculated by CDOCKER. The structural dynamics at 300 ns-trajectories under 297 and 310 Kelvin were analyzed and results showed that the complex at 310 K (3.03 Å) was slightly more fluctuated than that of the 297 K (2.91 Å) (Supplementary 2). The binding free energy (ΔG bind) was calculated from the equilibrium phase which occurred after 40 ns at both conditions. Results were subsequently compared to previously described (FN5Y)^21^ by SIE method (Fig. 5C). The binding free energy of cardol triene to the K and K’ sites were −9.41 ± 0.03, and −8.45 ± 0.03 kcal/mol at 297 K; and −9.29 ± 0.04, and −8.87 ± 0.07 kcal/mol at 310 K, respectively. Interestingly, the energy from cardol triene/DENV E complex were significantly higher than that of the previously reported fusion inhibitor, FN5Y at −3.96 ± 0.03 and −4.30 ± 0.03 kcal/mol at K and K’ sites, respectively, with *p*-value = 0.0026^21^. In addition to SIE, MM-GB/PBSA and QM/MM-GBSA (SCC-DFTB) methods were used to corroborate the binding energy of cardol triene and the kl hydrophobic pockets. The binding energy was achieved from 200 ns-trajectories and delicately computed at K and K’ sites at 297 and 310 K using previously described methods (Fig. 5D). Results showed that cardol triene consistently bound to K and K’ sites regardless of the binding free energy calculations or temperature conditions. The major reason that cardol triene contributed to a significantly tight binding was the additional hydrophobic interactions from the hydrocarbon chain and the kl hydrophobic pocket^22,23^. Major binding residues were revealed and grouped into four regions as follows; 48–54 (T48, E49, A50, P53), 128–135 (K128, V130, L135), 191–207 (M196, L198, Q200, W206, L207), and 270–281 (I270, and L277) (Fig. 5E). Noted that there were multiple hydrophobic interactions at A50, L135, M196, L198, W206, L207, I270 and L277 residues (Fig. 5E). However, the major ligand-binding interaction was contributed by polar and charged residues at T48, E49, K128 and Q200 (Fig. 5E)^22–24^.

**Figure 6:**
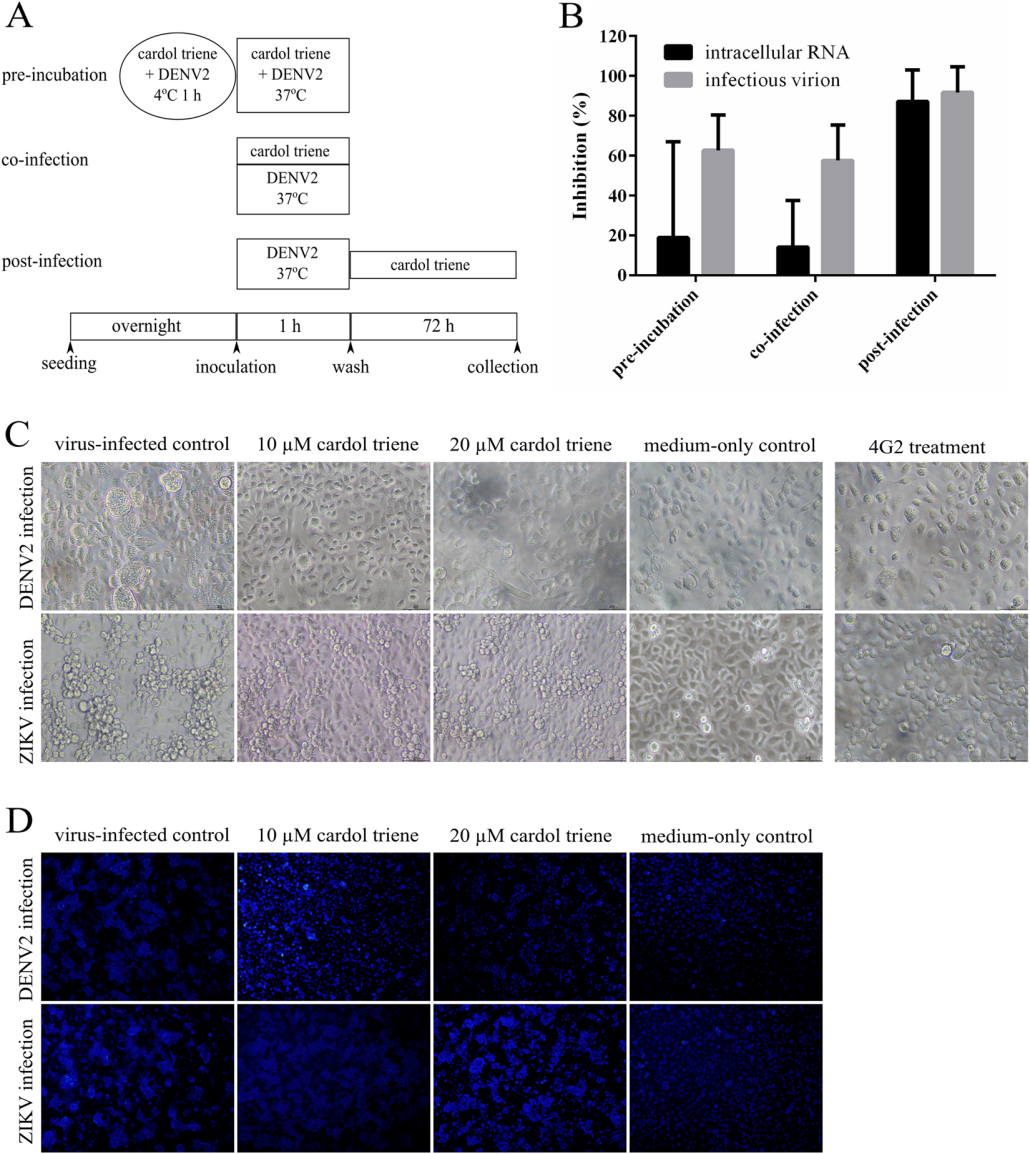
Cell-based functional assays. (**A**,**B**) Attachment inhibition study was performed as illustrated in (**A**) diagram. (**B**) Cell lysates and culture supernatants were analyzed by RT-qPCR and plaque titrations and calculated as percent of DMSO-treated cells for DENV2 intracellular RNAs and infectious virions, respectively. (**C**,**D**) Fusion inhibition study of DENV2 and ZIKV. C6/36 cells were infected with the virus at the M.O.I. of 1 for 1 h with gentle rocking. Cardol triene at 10 and 20 µM, and 4G2 antibody were added to the virus-infected cells. Cells were washed and maintained in culture medium in 28 °C for 48 h. MES were added to induce the acidic pH mimicking endosomal pH-dependent fusion. Pictures were taken when fused cells (**C**) or nuclei (**D**) were observed under light microscope or DAPI staining, respectively. Results were confirmed by three independent experiments.

Since dengue envelope protein was the key factor to receptor-binding, endocytosis, and fusion, we asked the question whether the compound would mainly neutralize the virion, inhibit the receptor binding, or inhibit endosomal fusion. Cardol triene at 10 µM was then co-incubated with DENV2 before, during, and after infection to Vero cells (Fig. 6A). Results showed that the major inhibition of intracellular RNA and infectious virion was observed at post-infection at 87.00 ± 6.43% and 91.73 ± 4.53% (Fig. 6B), respectively. Therefore, the major target was most likely at a pH-dependent endosomal fusion. Moreover, moderate inhibition was also noticed in infectious virions during pre-incubation and co-infection, suggesting that the compound also moderately neutralized the virion and interfered with the receptor-binding. However, the major post-infection inhibition required further investigations.

Fusion inhibition study^25–27^ of DENV and ZIKV were used to confirm the findings. Briefly, cardol triene at 10 µM was added to DENV2 or ZIKV (Fig. 6C-D) infected C6/36 cells at the M.O.I. of 1 for 48 h before inducing acidic condition by MES. The 4G2 antibody was used as a positive control inhibiting both DENV2 and ZIKV fusion^28^. Results showed that cardol triene exclusively inhibited dengue fusion as seen in the unfused cells and nuclei (Fig. 6C-D). ZIKV-induced fused cells and nuclei were present in cardol triene-treated samples (Fig. 6C-D). Therefore, we concluded that one of the targets of cardol triene, and other CNSL-derived phenolic lipids, was the kl loops of DENV E protein, thus preventing the conformational change of DENV dimer to trimer that was required to initiate pH-dependent fusion.

### Cardol triene inhibited late stages of DENV replication

**Figure 7:**
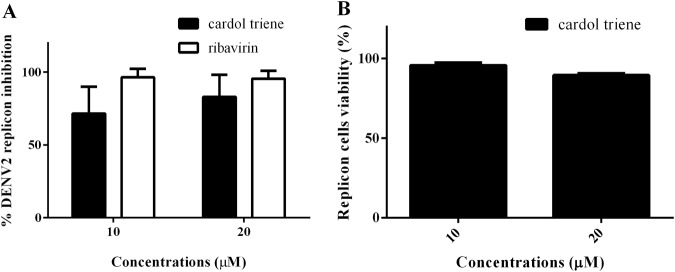
Replicon inhibition study. (**A**) DENV2/BHK-21 replicon (5 × 10^4^ cells) were treated with cardol triene or ribavirin for 72 h. Percent inhibition was calculated from RT-qPCR of cell lysate. (**B**) Percent DENV2/BHK-21 replicon cell viability at 72 h under 10 and 20 µM cardol triene. Error bars represented standard error of means (SEM) from three independent experiments.

We analyzed the replication inhibition using BHK-21/DENV2 replicon cells stably expressed the non-structural proteins^44^. Cardol triene at 10 µM and 20 µM were added to the replicon cells for 72 h and the replication inhibition was accessed by RT-qPCR of DENV2 NS1. Ribavirin, a known inhibitor of flaviviral replication, was used as a positive inhibition control^29^. We found 76.60 ± 7.59 and 89.44 ± 4.02 percent DENV2 RNA inhibitions from 10 µM and 20 µM cardol triene, respectively (Fig. 7A), comparable to those of ribavirin. The inhibitory effect at 20 µM of cardol triene was closed to ribavirin that showed 98.42 ± 0.68 percent and 98.09 ± 0.72 percent at 10 µM and 20 µM, respectively. Replicon cell viability under cardol triene treatment was also analyzed using an MTS assay. Cardol triene treatment at 10 µM and 20 µM for 72 h did not have effect on cell viability (Fig. 7B). Therefore, we concluded that cardol triene inhibited DENV RNA replicon replication, potentially in dose-dependent manner.

## Discussion

Phenolic lipids were reported as active ingredients of plants in the *Family* *Anacardiaceae* with versatile biological activities^30^. Major components of phenolic lipids in this study were directly isolated from cashew nut shell liquid (CNSL) and synthesized by hydrogenation. The viral inhibition was analyzed and cardol triene was the most effective compound in this group considering from the highest CC_50_ and TI values. Cardol triene was also similarly effective against the other DENV but no inhibition was found in Zika virus. Moreover, cardol triene was observed with high CC_50_ values in three human-derived cell lines; HepG2, HEK-293, and THP-1. One of the molecular targets was the kl hydrophobic pockets that would undergo conformational change under acidic pH and performed endosomal fusion to release the viral genome. Our findings showed for the first time that CNSL-derived phenolic lipids had a potential for further development as anti-dengue inhibitors.

Since all homologues showed similar efficacies against DENV2 at the kl loops (Table 1, Fig. 5A), we then hypothesized that all CNSL-derived phenolic lipids shared the same molecular targets. The major target of cardol triene, and potentially to other phenolic lipids, was the kl loop of DENV E protein. This target was one of the hotspots in dengue drug discovery and a variety of ligands were reported as fusion inhibitors^22,23,26,31,32^. Cardol triene was structurally similar to β-OG, the crystalized ligand, and similar binding moieties were identified. Additional structure-activity relationship (SAR) study should focus on optimizing the phenolic lipids into this binding pocket. A previous report on mannoside glycolipid conjugates suggested that the C24 hydrocarbon tail, either saturated or unsaturated, was active DENV2 inhibitor^33^, whereas the shorter C21 and C19 did not show any inhibition. Therefore, it is interesting to explore whether the tail length (C15-C25), and level of unsaturation would reflect the binding efficiency. Further investigations should also include biophysical experiments such as isothermal titration calorimetry (ITC) or surface plasmon resonance (SPR) to verify the MD simulation results. Moreover, saturated anacardic acid (C15:0), which inhibited DENV2 at the EC_50_ of 4.82 ± 1.71 µM (Table 1), was previously reported to inhibit another member of *Flaviviridae* family, hepatitis C virus (HCV), with the EC_50_ of 7.25 µM^19^. However, cardol triene did not inhibit ZIKV although the virus was closely related to DENV. Based on the findings, we speculated that the similar efficacies of DENV and HCV might be a coincidence of the compound targeting different molecules. Indeed, the viral inhibition (EC_50_) was a summary of hundreds of viral activities and virus-host interactions.

Cardol triene showed cytoprotective effects towards Vero, HepG2, THP-1, and HEK-293 cell lines with the CC_50_ of 207.3 ± 5.24, 140.27 ± 8.44, 129.77 ± 12.08, 92.8 ± 3.93 µM, respectively (Fig. 3A–C). The cytoprotective effect of cardol triene was also reported in normal human lung fibroblast cell line, GM07492A, with the 192.6 ± 6.0 µM^34^. Cardol monoene, however, was reported with a cytotoxicity against the SW620 (CC_50_ of 14.18 ± 0.76 µM), KATO-III (CC_50_ of 19.06 ± 0.39 µM), HepG2 (CC_50_ of 2.23 ± 0.22 µM), Chago I (CC_50_ of 2.55 ± 0.18 µM) and BT474 (CC_50_ of 13.46 ± 0.14 µM) cell lines^16,18,35^. Also, the saturated anacardic acid was cytotoxic against K562 and AGS cell lines with the CC_50_s of 25.4 µM and 41.6 µM, respectively^36^. Therefore, the saturated and monoene compounds were suitable for further development as anti-cancer chemotherapy. Moreover, we noticed that the increasing unsaturated hydrocarbon tails positively correlated to the higher CC_50_ values (Table 1). Previous reports showed that the longer and the more unsaturated hydrocarbon tail related to higher affinity to either disruptive and stabilizing the lipid bilayer^17,37–39^. It is possible that the increasing unsaturated hydrocarbon tail would facilitate the compound incorporation into lipid bilayer and stabilizing the bilayer by increasing the membrane fluidity^17^. Moreover, among the three head groups, cardol showed the highest cytoprotection possibly because cardol had a rod-like shape with adequate hydrophobicity for insertion and stabilization into the hydrophobic parts of bilayer. Anacardic acids had a conical shape and more hydrophilic than cardol, therefore it was incorporated into the subsurface and introduced a constraint on lipid packing leading to earlier membrane disruption^17,37^. We proposed that insertion and stabilization was a strategy of cardol triene to protect the cell membrane, thus tremendously increasing CC_50_s in all tested cell lines. In addition, a previous report suggested that the incorporation of phenolic lipids into lipid membrane could promote liposome fusion to the cell membrane and could activate the release of drug molecules from the interior of liposomes to the target cells^39^. In our case, cardol triene itself also actively inhibited DENV envelope fusion; therefore, the compound could possibly be delivered into DENV-inhabiting endosome via liposome fusion, which would subsequently bind to kl hydrophobic pockets as previously described.

Our results showed that DENV replicon RNA was inhibited by cardol triene (Fig. 7). The replicon is a self-replicating viral RNA utilized as a screening for RNA replication inhibition. However, the exact molecular target had not been elucidated. Viral replication is driven and modulated by hundreds of viral and host factors including translation machineries, lipid biosynthesis, chaperones, etc. Previous reports showed that saturated anacardic acid inhibited host-derived histone acetyltransferase (HATs), inhibited IL-8 production^40^ and HCV replication^19^. However, more evidence is required in order to conclude that dengue virus use the same inhibitory platform. Investigating the compounds with similar constructs can be further employed by similarity search and analysis of their biological activities could lead to the possible molecular target. Note that the inhibition was limited only to DENV so the targets would also be narrowed down to factors involving in DENV replication.

In conclusion, we demonstrated for the first time that phenolic lipids, especially cardol triene, were potential candidates for drug development. The compound inhibited all serotypes of DENV with good efficacy and mild cytotoxicity. One of the molecular targets were identified at kl loops of E protein at the early stage. The structure-activity relationship study of phenolic lipids and the kl loops should be further explored for potentials to be antiviral chemotherapy. Moreover, its selective efficacy towards DENV but not to ZIKV could become a crucial tool for structural and functional comparative studies of these viruses. Since this group of compounds are relatively new in antiviral drug discovery, a lot of knowledge is awaited to be explored and discovered. And we clearly showed in this research that cardol triene has strong potentials to become novel antivirals.

## Materials and Methods

### Extraction, Purification, and Identification of Anacardic acids, Cardanols, and Cardols

Phenolic lipids were extracted from cashew nut shell liquid (CNSL) (*Anacardium occidentale)*, purified according to a previously reported protocol^41^ and characterized by ^1^H NMR (CDCl_3_) spectroscopy. The products were analyzedas anacardic acid, cardanol, and cardol, 84, 2, and 0.1 percent, respectively. Briefly, 5% aqueous MeOH (300 mL) (Sigma Aldrich^®^, St. Louis, USA) was used to dissolve CNSL (50 g). Ca(OH)_2_ (50 g) (Sigma Aldrich^®^, St. Louis, USA) was gradually added to the reaction mixture while stirring at 50 °C for 5 h. The reaction was monitored by Kieselgel 60 PF_254_ silica-based thin layer chromatography (TLC) (Merck, New Jersey, USA). After 5 h reaction, dark brown calcium anacardate was precipitated which was then filtered and washed with MeOH before drying under vacuum evaporator. The product was suspended in HCl solution (Sigma Aldrich^®^, St. Louis, USA) and continuously stirred for 1 h before extraction with EtOAc (Sigma Aldrich^®^, St. Louis, USA). The organic layer was collected and washed with water, and subsequently dried over anhydrous Na_2_SO_4_ (Sigma Aldrich^®^, St. Louis, USA). Finally, the product was concentrated under reduced pressure to yield anacardic acid (42.1 g, 84% yield). The leftover filtrates from calcium anacardate precipitation was also evaporated under reduced pressure and subsequently extracted using EtOAc. The organic layer was recovered and dried over anhydrous Na_2_SO_4_ before concentration under reduced pressure. The crude product was further purified by silica column with hexane/EtOAc elution to cardanol (1.1 g, 2% yield) and cardol (319.1 mg, 0.1% yield). Each species was further separated into pure homologues of saturated (C15:0), monoene (C15:1), diene (C15:2), and triene (C15:3) using semi-preparative high performance liquid chromatography. The characteristics and signals of all compounds were shown as follows;

Anacardic acid C15:0 (6-pentadecylsalicylic acid): white solid (18% of total anacardic acids) ^1^H NMR(CDCl_3_) δ_H_ (ppm): 11.01 (1 H, s), 7.36 (1 H, t, J = 7.9 Hz), 6.87 (1 H, dd, J = 8.3, 1.2 Hz), 6.78 (1 H, dd, J = 7.5, 1.2 Hz), 2.98 (2 H, t, J = 8.0 Hz), 1.59 (2 H, m), 1.26 (19 H, m), and 0.88 (3 H, t, J = 6.8 Hz).

Anacardic acid C15:1 (6-[8(Z)-pentadecenyl]salicylic acid): yellow liquid (31% of total anacardic acids) ^1^H NMR (CDCl_3_) δ_H_ (ppm): 11.04 (1 H, s), 7.35 (1 H, t, J = 7.9 Hz), 6.86 (1 H, d, J = 8.3 Hz), 6.77 (1 H, d, J = 7.5 Hz), 5.35 (2 H, m, J = 4.8 Hz), 2.97 (2 H, t, J = 7.9 Hz), 2.01 (4 H, m), 1.60 (2 H, m), 1.29 (16 H, m), and 0.88 (3 H, t, J = 6.5 Hz).

Anacardic acid C15:2 (6-[8(Z), 11(Z)-pentadecadienyl]salicylic acid): yellow liquid (26% of total anacardic acids) ^1^H NMR (CDCl_3_) δ_H_ (ppm): 11.07 (1 H, s), 7.35 (1 H, t, J = 7.9 Hz), 6.86 (1 H, d, J = 8.3 Hz), 6.77 (1 H, d, J = 7.5 Hz), 5.37 (4 H, m), 2.97 (2 H, t, J = 7.9 Hz), 2.77 (2 H, t, J = 6.3 Hz), 2.01 (4 H, m), 1.33 (12 H, m), and 0.90 (3 H, t, J = 7.4 Hz).

Anacardic acid C15:3 (6-[8(Z), 11(Z), 14-pentadecatrienyl]salicylic acid): yellow liquid (25% of total anacardic acids) ^1^H NMR (CDCl_3_) δ_H_ (ppm): 11.09 (1 H, s), 7.35 (1 H, t, J = 7.9 Hz), 6.86 (1 H, d, J = 8.3 Hz), 6.76 (1 H, d, J = 7.5 Hz), 5.81 (1 H, m), 5.39 (4 H, m), 5.01 (2 H, m), 2.97 (3 H, t, J = 7.9 Hz), 2.79 (4 H, dd, J = 13.7, 7.5 Hz), 2.05 (2 H, m), 1.59 (2 H, m), and 1.34 (8 H, m).

Cardanol C15:1 (3-[8(Z)-pentadecenyl]phenol): yellow liquid (31% of total cardanols) ^1^H NMR (CDCl_3_) δ_H_ (ppm): 7.14 (1 H, t, J = 7.7 Hz), 6.75 (1 H, d, J = 7.6 Hz), 6.65 (2 H, d, J = 8.4 Hz), 5.35 (2 H, m), 2.55 (2 H, t, J = 7.8 Hz), 2.01 (4 H, m), 1.59 (2 H, m), 1.29 (16 H, m), and 0.89 (3 H, t, J = 6.5 Hz).

Cardanol C15:2 (3-[8(Z), 11(Z)-pentadecadienyl]phenol): yellow liquid (46% of total cardanols) ^1^H NMR (CDCl_3_) δ_H_ (ppm): 7.14 (1 H, t, J = 7.7 Hz), 6.75 (1 H, d, J = 7.5 Hz), 6.65 (2 H, d, J = 8.1 Hz), 5.36 (4 H, m), 2.78 (2 H, t, J = 6.3 Hz), 2.55 (2 H, t, J = 7.8 Hz), 2.04 (4 H, m), 1.58 (12 H, m), and 0.91 (3 H, t, J = 7.4 Hz).

Cardanol C15:3 (3-[8(Z), 11(Z), 14-pentadecatrienyl]phenol): yellow liquid (23% of total cardanols) ^1^H NMR (CDCl_3_) δ_H_ (ppm): 7.14 (1 H, t, J = 7.6 Hz), 6.76 (1 H, d, J = 7.6 Hz), 6.65 (2 H, d, J = 7.9 Hz), 5.83 (1 H, m), 5.40 (4 H, m), 5.03 (2 H, m), 2.82 (4 H, dt, J = 16.1, 6.1 Hz), 2.56 (2 H, t, J = 7.7 Hz), 2.04 (2 H, m), 1.60 (2 H, m), and 1.29 (8 H, m).

Cardol C15:2 (5-[8(Z), 11(Z)-pentadecadienyl]resorcinol): brown liquid (73% of total cardols) ^1^H NMR (CDCl_3_) δ_H_ (ppm): 6.24 (2 H,s),6.17 (1 H, s),5.36 (4 H,m, J = 8.1, 4.8 Hz), 2.78 (2 H, t, J = 6.3 Hz), 2.48 (2 H, t, J = 7.8 Hz), 2.04 (4 H, m), 1.56 (2 H, m), 1.33 (8 H, m), and 0.91 (3 H, t, J = 7.4 Hz).

Cardol C15:3 (5-[8(Z), 11(Z), 14-pentadecatrienyl]resorcinol): brown liquid (27% of total cadanols) ^1^H NMR (CDCl_3_) δ_H_ (ppm): 6.25 (2 H, s), 6.17 (1 H, s), 5.82 (1 H, m), 5.39 (4 H, m), 5.02 (2 H, m), 2.80 (4 H, dt, J = 14.1, 7.9 Hz), 2.46 (2 H, t, J = 7.7 Hz), 2.04 (2 H, m), 1.55 (2 H, m), and 1.28 (8 H, m).

In addition, the saturated homologues (C15:0) of cardanol and cardol were synthesized from hydrogenation reaction and their signals were shown as follows;

Cardanol C15:0: ^1^H NMR (CDCl_3_) δ_H_ (ppm): 7.15 (1 H, t, J = 7.7 Hz), 6.77 (1 H, d, J = 7.6 Hz), 6.68 (2 H, d, J = 8.4 Hz), 5.91 (1 H, s), 2.56 (2 H, t, J = 7.9 Hz), 1.60 (2 H, t, J = 7.6 Hz), 1.30 (24 H, m), 0.91 (3 H, t, J = 6.6 Hz).

Cardol C15:0: ^1^H NMR (CDCl_3_) δ_H_ (ppm): 6.24 (2 H, s), 6.18 (1 H, s), 2.47 (2 H, t, J = 7.8 Hz), 1.55 (2 H, m), 1.25 (24 H, m), 0.87 (3 H, t, J = 6.6 Hz).

All compounds were stored as solid powder at room temperature. The compounds were prepared in dimethyl sulfoxide (DMSO) (Sigma Aldrich®, St. Louis, USA) to 50 mM stock solutions and stored as aliquots at −20 °C until use.

### Cells and viruses

Vero (ATCC^®^CCL-81), LLC/MK2 (ATCC^®^ CCL-7), C6/36 (ATCC^®^ CRL-1660), HEK-293 (ATCC^®^ CRL-1573), HepG2 (ATCC^®^ HB-8065), and THP-1 (ATCC^®^ TIB-202) cells were maintained as previously described^42^. Also, DENV1 (16007), DENV2 (New Guinea C strain), DENV3 (16562), DENV4 (c0036), and Zika virus (SV0010/15) reference strains were propagated in Vero and C6/36 cells as previously described^42^.

### Efficacy studies

The efficacies of anacardic acid, cardol, and cardanol mixtures were primarily studied using DENV2 infected Vero cells. Vero (5 × 10^4^) cells were seeded into each well of 24-well plates. After an overnight incubation, cells were infected with DENV2 (M.O.I. of 0.1) and 10 µM and 25 µM phenolic lipids. DMSO at 1% was used as a mock treatment with 100% infectivity. The compounds were co-incubated with cells 37 °C for 3 days before supernatants collection. Viral titers were evaluated by plaque titration^43^.

Next, each homologue was analyzed for effective concentration (EC_50_). Vero (5 × 10^4^) cells were seeded, and infected with DENV2 (M.O.I. of 0.1) as previously described^42^. Phenolic lipid mixtures and homologues were serially diluted in DMSO to the final concentrations as follows; 0, 0.5, 1, 1.5, 2, 2.5, 5, 7.5, 10, 25, and 50 µM. DMSO at the concentration of 1% was used as a mock treatment referring to 100% infection. Infected cells were treated with the designated compounds during and post infection. Supernatants were collected at 3 days after infection for analysis of virion production by plaque titration. Non-linear regression analysis was used to determine the effective concentrations. Three independent experiments were performed and results were as means and standard error of the means (SEM). The therapeutic index (TI) was a ration of CC_50_/EC_50_ of the compound.

### Toxicity studies

The primary screening was performed using Vero (10^4^) cells seeded into each well of 96-well plates. Phenolic lipids at 10 µM and 25 µM were added to the experimental wells after an overnight incubation. DMSO at 1% was used as a control referring to 100% cells viability. Cell viability was accessed at designated time-points using CellTiter 96® Aqueous One Solution Cell Proliferation Assay kit (Promega, Wisconsin, USA) and analyzed by spectrophotometer (*A*_450nm_) (VICTORTM X3, PerkinElmer, Massachusetts, USA). Results were calculated to percent cell death.

Cytotoxic concentrations (CC_50_) were also studied using Vero cells. Briefly, the 10^4^ cells were seeded into each well of 96-well plates. After an overnight incubation, phenolic lipids were serially diluted in DMSO to the final concentrations as follows; 25, 50, 100, 200, 300, 400, and 500 µM. Each concentration was performed in quadruplicates. DMSO at 1% was used as a mock treatment referring to 100% cell viability. Cells were incubated for 48 h before cell viability was accessed using CellTiler 96® Aqueous One Solution Cell Proliferation Assay kit (Promega, Wisconsin, USA). Non-linear regression analysis was used to determine cytotoxic concentrations (CC_50_) of each experiment. Results were derived from three independent experiments and reported as means and standard error of the means (SEM).

### Time of drug addition study (TOA)

Vero (5 × 10^4^) cells were seeded into each well of 24-well plates. cells were infected with DENV2 (M.O.I. of 0.1) after an overnight incubation^44,45^. Cardol triene (C15:3) at 10 and 20 µM were added to the cells during (0 h) and after infections at early time-points as follows; 1, 2, 4, 6, 8, 10, 12, 24, 36, and 48 h. Moreover, late time-points after were also accessed at 12, 14, 16, 18, 20, 22, 24, 36, 48, 60, and 72 h after infections. DMSO alone was added to the infected cells at respective time-points as a mock treatment referring to 100% infectivities. After 72 h incubation, cell lysates and supernatants were collected for analysis of intracellular RNAs and infectious virions by RT-qPCR and plaque titration, respectively. Results were confirmed by three independent experiments. Data were calculated, assembled, and reported as percent intracellular RNAs and infectious virions under the drug treatment at designated time-points.

### Attachment Inhibition study

Vero (5 × 10^4^) cells were seeded into 24-well plates as previously described. DENV2 (M.O.I. of 1) was diluted in ice-cold MEM with 1% fetal bovine serum^42^ and was allowed to adsorb on the cells for 1 h with continuous rocking. Cardol triene (10 µM) was prepared in DMSO and was mixed with DENV2 (M.O.I. of 1) before adsorption for 1 h (pre-attachment). Cardol triene (10 µM) was also mixed with DENV2 (M.O.I. of 1) and immediately applied to the cells (co-attachment). Last, cardol triene was added to the cells after 1 h DENV2 adsorption (post-attachment). The experiments were incubated for 2 days at 37 °C. Intracellular viral RNA was determined from cell lysate using RT-qPCR and infectious virion was determined using plaque titration of the supernatant. DMSO was added to the experiment as a no-inhibition control. Means and standard error of the means (SEM) of three independent experiments were noted.

### Fusion inhibition study

C6/36 (2 × 10^5^) cells were seeded into 24-well plates before infection with DENV2 and ZIKV (M.O.I. of 1) as described^42^. Cardol triene at 10 µM were introduced to the DENV2 and ZIKV infected cells during and after infection. DMSO was used as a 0% inhibition control and mouse anti-Flavivirus envelope protein antibody (4G2) was used as a 100% inhibition control. Cells were incubate at 28 °C for 2 days before addition of 0.5 M 2-(N-morpholino) ethanesulfonic acid (MES) (pH 5.0) (Sigma Aldrich, St Louis, USA). Moreover, cells from selected wells subsequently underwent DAPI staining. Cell-cell fusion was observed under brightfield and fluorescence using an Eclipse TS100 Inverted Routine Microscope (Nikon, New York, USA).

### Replicon inhibition study

BHK-21 (5 × 10^4^) cells expressing DENV-2 replicon (BHK-21/DENV2)^44^ were seeded as previously described. Cardol triene at 10 µM and 20 µM, DMSO, and ribavirin (TargetMol, USA) were added to the replicon cells and incubated at 37 °C for 3 days. Cells lysates were collected and viral RNA was quantified by RT-qPCR as previously described^44^. Data were reported as percent inhibition compared with ribavirin. Three biological replicates were performed and results were means and SEM of all replicates.

### Computational study

Phenolic lipids were evaluated for druglikeness by scoring their pharmacological characters (cLogP and solubility)^23,46^, and toxicity risks (mutagenicity, tumorigenicity, irritating effect, and reproductive effect) using Molecular Property Explorer and RTECS database.

To refine the screening result and construct the initial complex structure, the 3D DENV envelope protein (PDB code: 1OKE^47^) was obtained to calculate the interaction energy with phenolic lipids using CDOCKER software (Discovery Studio 2.5, Accelrys Inc., San Diego, USA). Each kl loop was assigned to 10 Å diameter according to n-octyl-beta-D-glucoside (β-OG) originally bound in the crystal structure^48^. All phenolic lipids were calculated for partial charge distribution and docked into kl loop with 100 replicas with random conformation of ligand. The best score predicted by CDOCKER interaction energy was selected to construct an initial complex structure for molecular dynamics (MD) simulation using 3 different methods; Solvated Interaction Energy (SIE), Molecular Mechanics-generalized Born/Poisson Boltzmann solvent accessible surface area (MM-GB/PBSA), and Quantum mechanics/molecular mechanics generalized Born surface area (QM/MM-GBSA) with density functional theory-based tight binding (SCC-DFTB) methods.

The MD simulations were conducted for cardol triene/DENV E protein complex with two temperature systems; 297 K (23.85 °C) and 310 K (36.85 °C) implemented with the GPU accelerated pmemd.cuda program in the AMBER16 package program^49,50^. Both 297 K and 310 K systems were simulated with explicit solvent TIP3P water molecule with 15 Å around protein surface. Periodic boundary condition was applied in this MD simulation. The protonation state of the ligand, cardol triene, was assigned by pKa calculation in MarvinSketch program^50^ and 2 atoms of Na^+^ were added to neutralize the electrical charge of DENV E dimer using PDB2PQR^51–53^. The 1,500 steps of steepest descents (SD) followed by 1,500 steps of conjugated gradients (CG) in the SANDER module by AMBER 16 were used to minimize the interference of water molecules and hydrogen atoms. The systems were subsequently brought up to 297 K and 310 K within 200 ps and was stabilized at the designated temperature until 300 ns. The MD trajectories were collected every 0.2 ps during 200–300 ns production phase^54–56^. The stability of the cardol triene/DENV E protein complex at equilibrium was analyzed for the global root-mean-square displacement (RMSD) of binding free energy using CPPTRAJ module. Snapshots, taken every 1 ns within 200–300 ns-MD trajectories, were used as materials for calculating total binding free energy using SIE, MM-GB/PBSA, and QM/MM-GBSA (SCC-DFTB) methods^57,58^. Snapshots were also used to identify the per-residue decomposition energy contribution by MM/GBSA method^59^.

### Ethical Approval

This project was approved by Ethical Review board, Faculty of Medicine, Chulalongkorn University (COE 021/2017 and IRB 375/60) as exempted in compliance with the International guidelines for human research protection as Declaration of Helsinki, The Belmont Report, CIOMS guideline, International Conference on Harmonization in Good Clinical Practice (ICH-GCP) and 45CFR 46.101(b).

### The level of bio-containment

We performed all pathogen-related experiments in bio-containment level 2. BSL-2 standard operating procedure was performed under the regulation of ISO15189 and ISO15190.

## Electronic supplementary material

Supplementary Information

## Data Availability

All data supporting the findings can be found in the results and supplementary sections.
