# Supplementary material for: Cardol triene inhibits dengue infectivity by targeting kl loops and preventing envelope fusion
Source: Sci Rep. 2018 Nov 9;8:16643. doi: 10.1038/s41598-018-35035-w (PMC6226472; doi:10.1038/s41598-018-35035-w)

**Cardol triene inhibits dengue infectivity by targeting kl loops and preventing envelope fusion.**

Parichat Kanyaboon^1,2+^, Thanaphon Saelee^1,2+^, Aphinya Suroengrit^3^, Kowit Hengphasatporn^4^, Thanyada Rungrotmongkol^4,5^, Warinthorn Chavasiri^6^, and Siwaporn Boonyasuppayakorn^1*^

^1^Applied Medical Virology Research Unit, Department of Microbiology, Faculty of Medicine, Chulalongkorn University, Bangkok, 10330, Thailand

^2^Medical Microbiology, Interdisciplinary Program, Graduate School, Chulalongkorn University, Bangkok, 10330, Thailand

^3^Graduate Program, Faculty of Medicine, Chulalongkorn University, Bangkok, 10330, Thailand

^4^Bioinformatics and Computational Biology Program, Graduated School, Chulalongkorn University, Bangkok, 10330, Thailand

^5^Structural and Computational Biology Research Group, Department of Biochemistry, Faculty of Science, Chulalongkorn University, Bangkok, 10330, Thailand

^6^Center of Excellence in Natural Products Chemistry, Department of Chemistry, Faculty of Science, Chulalongkorn University, Bangkok, 10330, Thailand

^+^These authors contributed equally to the work.

# *Correspondence to [siwaporn.b@chula.ac.th](mailto:siwaporn.b@chula.ac.th)

# Supplementary 1 Cell morphology after cardol triene treatment

**Supplementary 2 The root-mean-square displacement (RMSD) plot for all atoms of cardol triene binding to kl loop region**

# Supplementary 1 Cell morphology after cardol triene treatment

Vero (5x10^4^) cells were seeded and inoculated in 24-well plates as previously described. Cells were infected with DENV2 (M.O.I. of 1 and 5) for 1 h with gentle rocking. Cardol mixture and cardol triene at 10 µM were introduced to the DENV2 infected cells during and after infection. DMSO, 10 µM cardol mixture and 10 µM cardol triene alone were used as no virus control and incubated for 48 h. The cell morphology was observed after 2, 24 and 48 h incubation. Picture were taken using an Eclipse TS100 Inverted Routine Microscope (Nikon, New York, USA).


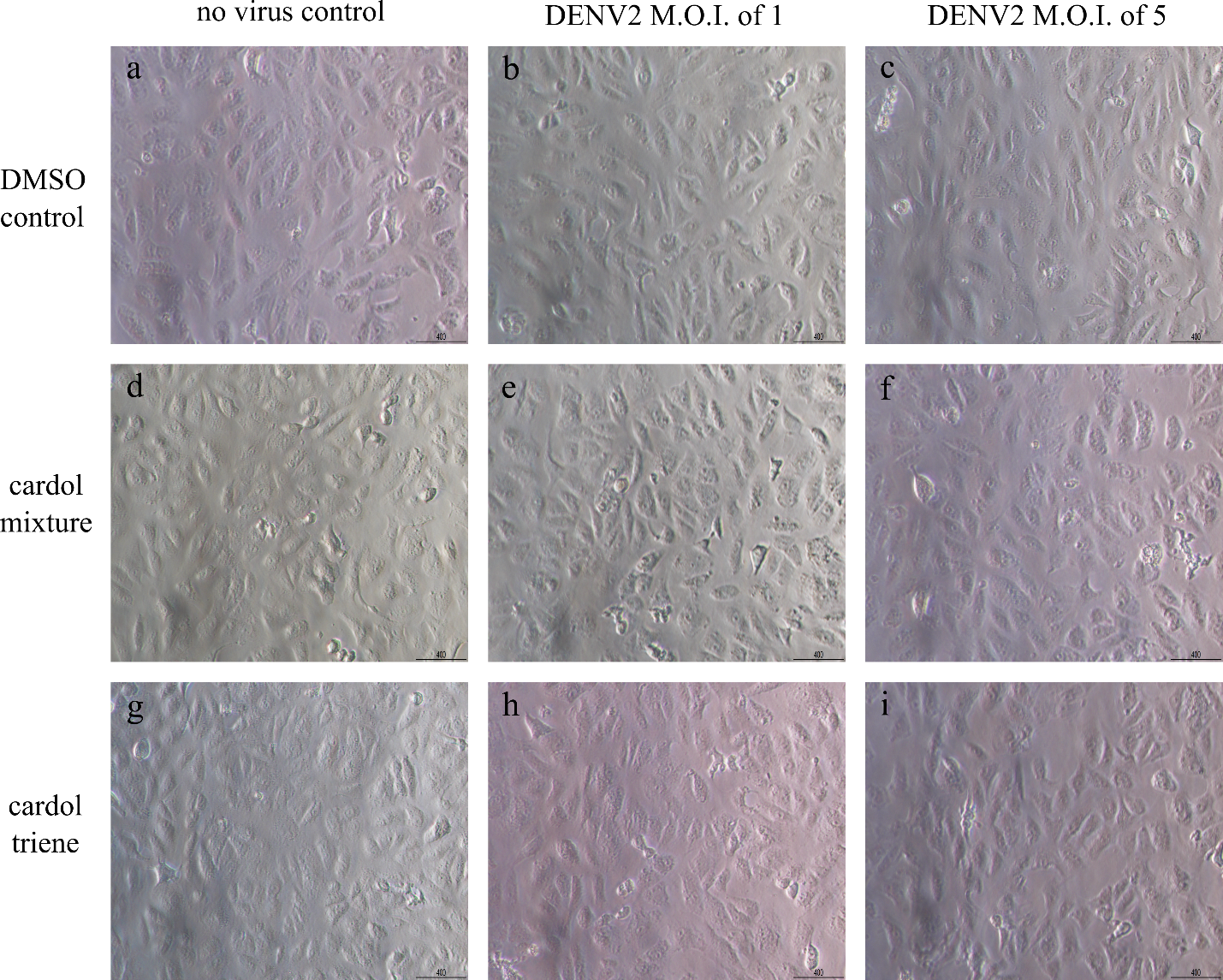


2 h


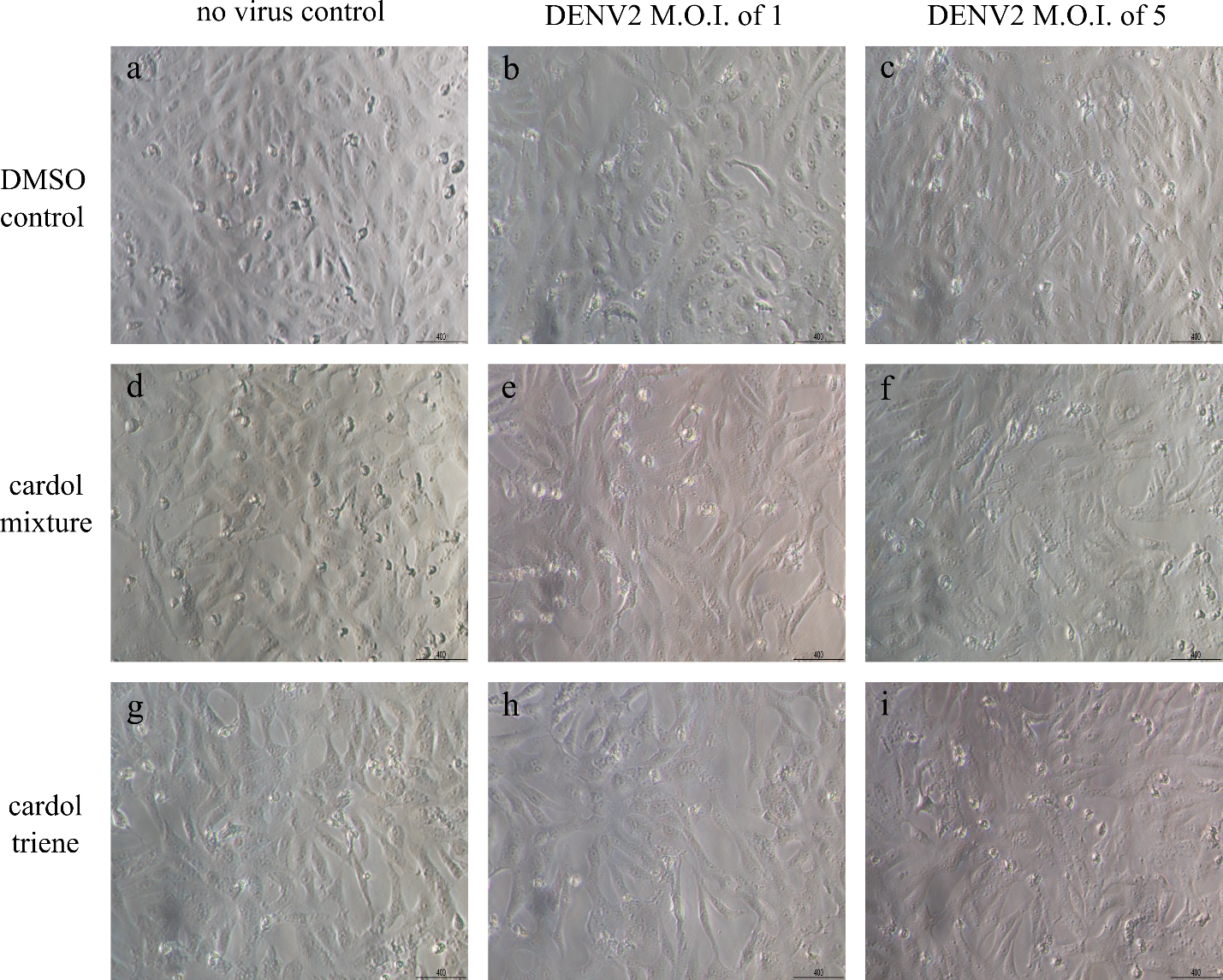

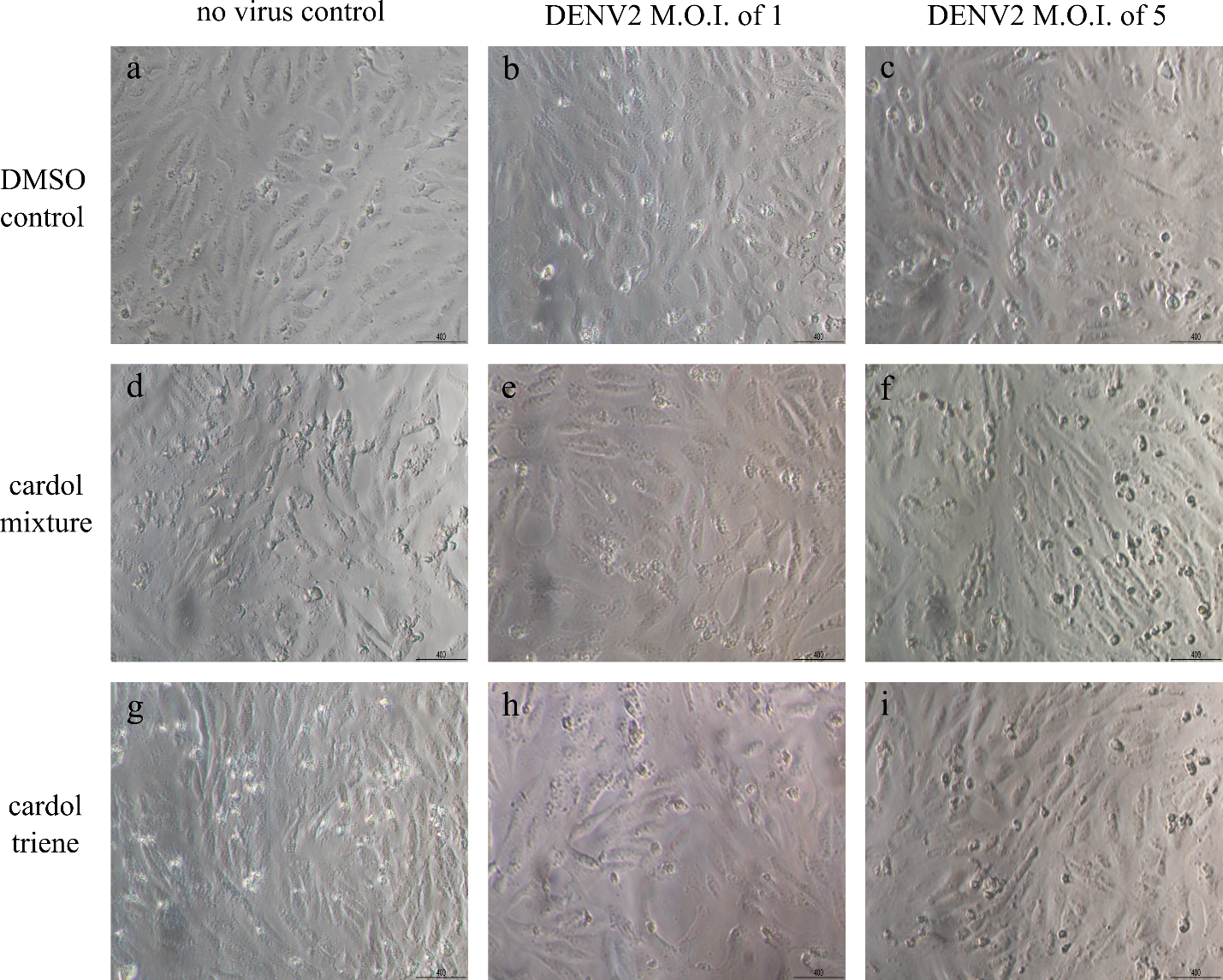


24 h

48 h

**Supplementary 2 The root-mean-square displacement (RMSD) plot for all atoms of cardol triene binding to kl loop region; K (blue) and K' (red), of DENV E protein (black and grey) at 297 and 310 Kelvin.**


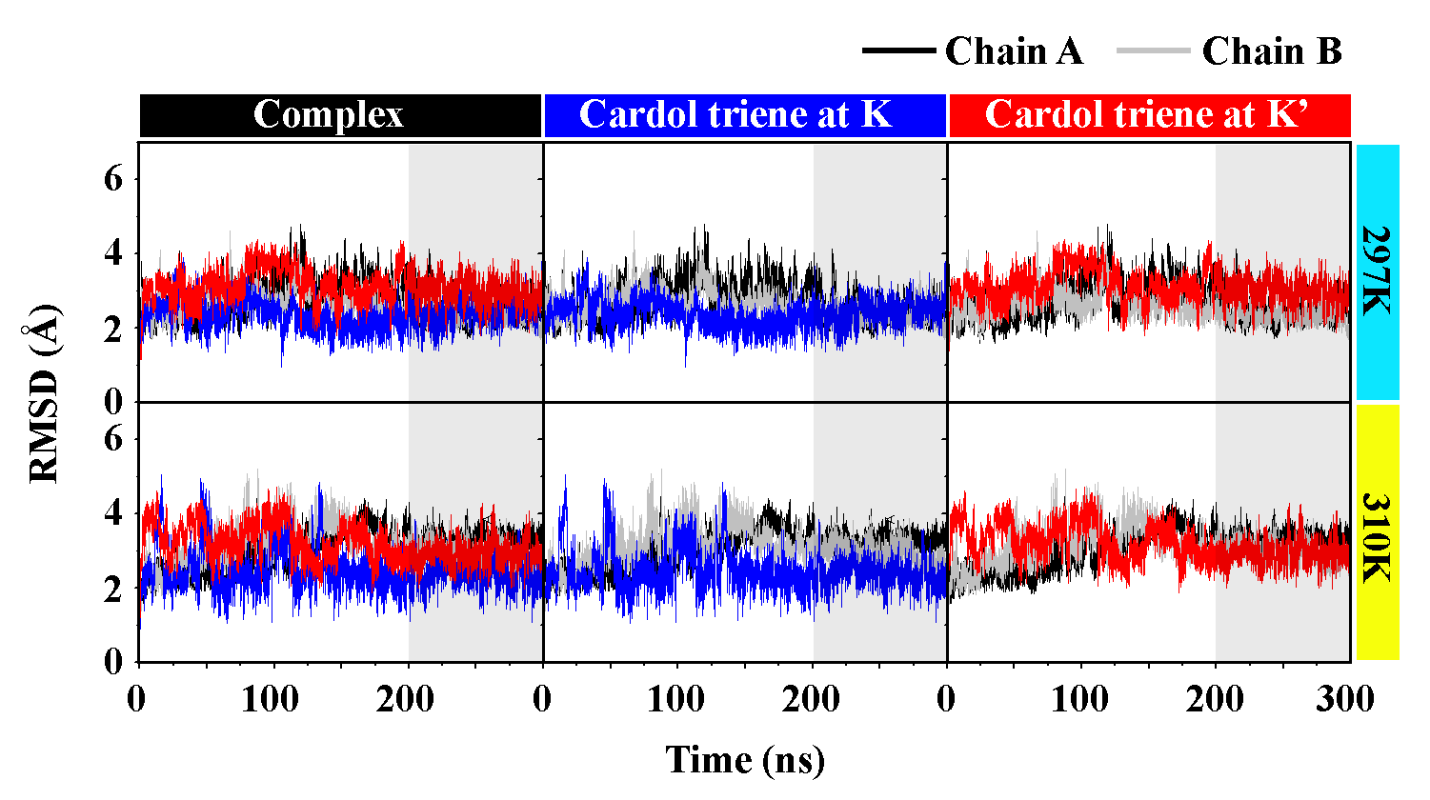

Supplement: Supplementary file 1 — Supplementary Information [file 41598_2018_35035_MOESM1_ESM.docx]
